# Theranostic Nanoparticles in Prostate Cancer: Disrupting Hypoxia‐Induced Glycolysis by Targeting Hypoxia‐Inducible Factor‐1 Alpha and Downstream Metabolites

**DOI:** 10.1002/cam4.71519

**Published:** 2026-01-11

**Authors:** Daniel Ejim Uti, Wilson Achu Omang, Esther Ugo Alum, Inalegwu Bawa, Okechukwu Paul‐Chima Ugwu, Ayodeji Oluwafemi Idowu, Item Justin Atangwho, Godwin Eneji Egbung

**Affiliations:** ^1^ Department of Biochemistry, Research and Publications Kampala International University Kampala Uganda; ^2^ Department of Biochemistry, Faculty of Basic Medical Sciences College of Medicine, Federal University of Health Sciences Otukpo Benue State Nigeria; ^3^ Department of Medical Laboratory Sciences College of Health Technology Calabar Nigeria; ^4^ Department of Environmental Sciences National Open University of Nigeria Abuja Nigeria; ^5^ Faculty of Basic Medical Sciences, Department of Biochemistry University of Calabar Calabar Nigeria

**Keywords:** glycolysis, HIF‐1α, hypoxia, metabolic reprogramming, prostate cancer, theranostic nanoparticles, tumor microenvironment

## Abstract

**Background:**

Prostate cancer (PCa) is a major cause of cancer‐associated death in men. A crucial factor in its development and treatment resistance is tumor hypoxia, which drives metabolic reprogramming (especially reconfiguration towards glycolysis), mediated to a great extent by hypoxia‐inducible factor‐“HIF‐1 alpha” (HIF‐1a).

**Aims:**

The present review summarizes (i) the mechanisms underlying hypoxia‐induced glycolysis that enhances the aggressiveness of and treatment failure in PCa and (ii) recent developments in the field of theranostic nanoparticles (TNPs) with dual actions of inhibiting HIF‐1a and downstream metabolic targets, while facilitating the imaging and treatment of the tumor.

**Materials and Methods:**

We summarize available evidence for the hypoxia‐glycolysis signaling in PCa and assess nanotechnology achievable theranostic approaches (i.e., liposomal‐, polymer‐ and metallic nanoplatforms) to promote drug delivery, real‐time tumor picture and modulation of hypoxic tumor microenvironments.

**Results:**

Hypoxia‐inducible factor‐1 alpha (HIF‐1a) driven hypoxia is a common phenotypic feature that underlies the increased glycolysis and aggressive tumor phenotype. TNPs have been developed with the aim of (a) enhancing the drug bioavailability, (b) enabling the selectivity of tumor and imaging, and (c) reducing the hypoxia‐linked metabolic pathways. The use of PCa as a model for TNP development is especially timely as hypoxia crosses the intersection of androgen receptor (AR) signaling heavens (hormone therapy resistance) leading to progression to castration‐resistant PCa (CRPC) and as the Prostate‐Specific Membrane Antigen (PSMA) is greatly overexpressed and is a validated target for custom imaging and treatment.

**Discussion:**

Compared with other hypoxia mediated solid tumors, hypoxia AR axis and PSMA overexpression have unique biological leverage for precision theranostics in PCa. Nevertheless, translation is limited by the issues of biocompatibility, complexities resulting from systematic regulations and constraints of scale‐up manufacturing.

**Conclusion:**

TNPs are a promising platform to integrate diagnosis and treatment of PCa as they incorporate features of targeted delivery, on‐line monitoring and interference with HIF‐1a regulated glycolysis. Future advances will require interdisciplinary optimization, development of better tumor‐targeting approaches, and artificial intelligence guided nanoparticle design to facilitate clinical scale up and regulation of technically and clinically acceptable theranostics of nanomedicines for PCa.

## Graphical abstract

The debate in the field of cancer immunotherapy is being changed as nanotechnology breaks the obstacle of cancer immune evasion, target delivery, and systemic toxicity. Esthetically advanced methods that are discussed in this review are nanovaccine, nanoparticle‐enabled checkpoint barrier, cytokine delivery, and T‐cell modulation techniques that augment immune activation and increase tumor selectivity. It also presents the current clinical progress and approved nano‐immunotherapeutics and highlights their safety and translatability. Collectively, these technologies place nanotechnology as an important determinant of the next‐generation, personalized cancer immunotherapy.

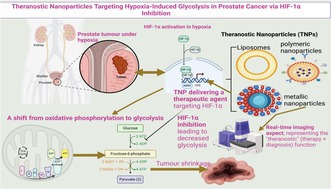

## 1 Introduction

The global proportion of prostate cancer (PCa) patients, a common form of male cancer, as reported by statistics, findings suggest that the number of new cases annually will rise from 1.4 million in 2020 to 2.9 million by 2040 [1], and new cases in 2025 is already at about 313,780 new cases of PCa, and about 35,770 deaths from PCa [2]. PCa develops because of various risk components, including age, heredity, ethnic background, lifestyle practices, and exposure to some environmental factors [3, 4]. African American males experience the most PCa cases along with the highest death rates, which indicates both genetic and social and healthcare system‐related factors play a role [5, 6]. The combination of obesity alongside high‐fat diets together with diminished physical activity levels creates conditions that raise the chances of developing PCa while speeding its progression [7, 8, 9].

PCa continues to stand as a substantial clinical challenge, though medical professionals have made progress in diagnosis and treatment. Patients with PCa can first get a radical prostatectomy, then radiation therapy along with androgen deprivation therapy (ADT), androgen antagonist therapy, and finally chemotherapy along with immunotherapy [10, 11]. The standard treatment modalities lead to various severe side effects, including urinary incontinence, sexual dysfunction, effects on the brain, depression, and an increased risk of cardiovascular problems. The primary challenge in dealing with PCa exists in the continuous development of castration‐resistant prostate cancer (CRPC). ADT lowers testosterone secretion from the testes and stops tumor growth, but most patients no longer benefit from it once they get CRPC, a type of aggressive PCa that is not responsive to treatment [12].

While hypoxia and metabolic reprogramming are observed in many solid tumors such as renal, pancreatic, and breast cancers, PCa presents several unique features that warrant its emphasis as the primary context of this review. First, PCa progression is intricately linked to androgen receptor (AR) signaling, and hypoxia‐driven pathways have been shown to synergize with AR suppression to promote therapy resistance and disease progression [13]. Second, the development of CRPC represents a distinctive clinical challenge where hypoxia and glycolytic shifts play a direct role in driving aggressive phenotypes [14]. Third, PCa uniquely offers a validated surface biomarker, prostate‐specific membrane antigen (PSMA), that is overexpressed in advanced disease and provides both diagnostic and therapeutic targeting opportunities for nanoparticles [14, 15]. Together, these features differentiate PCa from other hypoxia‐associated cancers and provide a strong rationale for selecting it as the central disease model for theranostic nanomedicine.

Researchers face hurdles in creating targeted treatments because different genetic and metabolic changes in PCa tumors drive their resistance to therapy [16, 17]. In the tumor microenvironment (TME) of solid tumors, including those that affect the prostate, hypoxia, which means lack of oxygen, is a key feature. As a tumor grows past the capacity of existing blood vessels, it creates areas with low oxygen levels [18, 19]. These areas cause metabolic and molecular changes that help cancer cells survive and invade while also increasing their ability to spread. Research has established that hypoxic regions in PCA tumors lead to unfavorable outcomes, including advanced disease characteristics and reduced responses to standard treatment methods. Better adaptable cells thrive in hypoxic conditions, which leads to the proliferation of more malignant cellular phenotypes [19, 20].

Hypoxia turns on hypoxia‐inducible factors (HIFs), mostly HIF‐1α and HIF‐2α [21]. These factors raise the levels of pro‐angiogenic factors, such as vascular endothelial growth factor (VEGF), which helps with angiogenesis. Even when oxygen is plentiful, PCa cells switch from oxidative phosphorylation (OXPHOS) to anaerobic glycolysis when they do not get enough oxygen (Warburg effect) [22, 23, 24]. This change in metabolism helps tumors stay alive by making them use more glucose and produce more lactate, which are both needed for important biosynthetic pathways in growth [24]. Scientists believe that hypoxia‐mediated glycolysis could serve as an effective treatment for cancer cells activated by low oxygen levels [25, 26]. When the body does not get enough oxygen, it produces more enzymes like hexokinase 2 (HK2), phosphofructokinase‐1F (PFK1), and lactate dehydrogenase A (LDHA). These enzymes aggravate PCa even after chemotherapy [27, 28]. It might be possible to make current treatments better by focusing on the specific metabolic pathways that are involved in tumors because they might affect the tumor's energy supply.

Theranostic nanoparticles (TNPs) are multifunctional nanocarriers designed to integrate diagnostic and therapeutic capabilities within a single platform, offering a promising approach for personalized PCa management [29, 30]. These biocompatible nanoparticles are commonly composed of lipids, polymers, silica, metals, or carbon‐based materials, each chosen for their physicochemical properties and compatibility with biological systems [31, 32, 33]. Their ability to facilitate real‐time tumor imaging alongside site‐specific drug delivery enhances treatment precision and minimizes systemic toxicity [34]. A key therapeutic strategy in PCa involves targeting the PSMA, a transmembrane protein overexpressed on malignant prostate cells [35]. TNPs functionalized with ligands, antibodies, or aptamers have demonstrated enhanced selectivity for PSMA, leading to improved therapeutic index and reduced off‐target effects [36]. For instance, studies have shown that PSMA‐targeted nanoparticles improve intracellular drug accumulation in PCa cells compared with non‐targeted systems, highlighting their role in overcoming traditional delivery barriers [37, 38].

PSMA‐targeted drug delivery systems may surmount conventional obstacles, providing enhanced cytotoxic efficacy and decreased systemic toxicity [39]. PSMA‐targeted dendrimers have shown selective tumor targeting in vivo and expedited clearance from non‐target organs [38]. Numerous PSMA‐targeted nanocarriers, including drug‐ligand conjugates, polymer nanoparticles, and liposomes, have been investigated to enhance the effectiveness of PCa therapies [40]. Therapeutically, TNPs have been employed to encapsulate chemotherapeutic agents such as docetaxel, paclitaxel, and doxorubicin. These nanocarriers enhance drug bioavailability and facilitate controlled release specifically at the tumor site, thereby reducing systemic toxicity [41]. For example, PEGylated liposomal nanoparticles have been reported to extend circulation time and evade immune detection, resulting in increased tumor accumulation and selective cytotoxicity against PCa cells, sparing adjacent healthy tissues [42, 43, 44].

In addition to chemotherapy, TNPs have shown efficacy in alternative therapeutic modalities. In photothermal therapy (PTT), gold and carbon‐based nanoparticles absorb near‐infrared light to produce localized hyperthermia, selectively inducing apoptosis in PCa cells [45, 46]. Likewise, in photodynamic therapy (PDT), TNPs are engineered to deliver photosensitizers that, upon light activation, generate reactive oxygen species (ROS) capable of selectively destroying cancerous tissue with minimal damage to normal prostate cells [47, 48]. These findings collectively underscore the versatility of TNPs in mediating multimodal treatment strategies.

RNA interference (RNAi), when integrated with gene therapy strategies, has been shown to enhance the efficacy of PCa treatment by enabling the targeted delivery of small interfering RNAs (siRNAs) and microRNAs (miRNAs) to downregulate specific oncogenes implicated in tumor progression [49]. Studies have demonstrated that siRNA‐mediated silencing of genes such as *AR*, B‐cell lymphoma 2 (*BCL2*), and Enhancer of Zeste Homolog 2 (*EZH2*), as well as miRNA modulation (e.g., restoration of tumor‐suppressive miRNAs like miR‐34a or inhibition of oncogenic miRNAs like miR‐21), significantly inhibits tumor cell proliferation, induces apoptosis, and improves therapeutic outcomes [50, 51, 52, 53]. Immunotherapy reaches its goal through immune checkpoint inhibitor‐functionalized nanoparticles that enhance vaccine delivery as well as antitumor immune responses [54, 55, 56]. While numerous preclinical studies support the potential of TNPs in PCa management, critical gaps remain in translating these platforms to clinical application. Future research must address challenges such as nanoparticle biodistribution, long‐term safety, and regulatory approval [57, 58, 59]. A deeper understanding of these factors will facilitate the development of clinically viable TNPs that fulfill the promise of precision nanomedicine in PCa.

Further integration with emerging insights into PCa biology highlights novel hypoxia‐associated and metabolic mechanisms. For instance, recent high‐impact studies demonstrate that: (1) the HIF1α–prolyl hydroxylase domain‐containing protein 1 (PHD1)–forkhead box protein A1 (FOXA1) axis orchestrates hypoxic reprogramming and suppresses AR signaling [60, 61]; (2) dysregulation of N6‐methyladenosine (m6A) RNA modification under hypoxic conditions links epitranscriptomic alterations to PCa aggressiveness [62]; and (3) investigations into the hypoxia/glycolysis framework provide mechanistic evidence [63, 64], supporting the concept that targeted nanoparticle systems (TNPs) could be rationally engineered to interfere with these pathways.

While nanomedicine has demonstrated broad applications in oncology, this review is structured to emphasize PCa as the central disease model. General discussions of nanomedicine principles are presented only to contextualize their translation into PCa therapy. By doing so, the manuscript highlights disease‐specific challenges such as castration resistance, PSMA‐targeting strategies, and hypoxia‐driven metabolic reprogramming, ensuring relevance to PCa researchers and clinicians. Accordingly, this review focuses specifically on PCa, emphasizing PSMA‐targeted delivery, strategies to overcome castration resistance, and the integration of nanoparticles with ADT, poly(ADP‐ribose) polymerase (PARP) inhibitors, and immunotherapy.

## 2 Hypoxia Biology and Metabolic Vulnerabilities in Prostate Cancer

### 2.1 Mechanisms of Hypoxia in Tumor Growth

The essential feature of TMEs known as hypoxia plays a crucial role in the progression of cancer along with metastasis and confers resistance to therapy [55, 65]. The expansion of tumors triggers insufficient oxygen supply which creates regions without sufficient oxygen [66]. The cells within tumors evolve survival mechanisms by undergoing three major changes during oxygen‐deficient states that boost their survival rates and aggressive behavior [66].

### 2.2 Oxygen Deprivation and Metabolic Adaptation

#### 2.2.1 Tumor Hypoxia and Oxygen Consumption

The excessive demand for oxygen which exceeds the supply occurs in solid tumors because uncontrolled cell division advances at a rate that exceeds blood vessel oxygen delivery capacity [67]. Oxygen supply imbalance in solid tumors results in the formation of three distinct regions: normoxic (well‐oxygenated) regions, hypoxic (oxygen‐deficient) regions, and necrotic (oxygen‐deprived, dead cell) regions. The quantity of available oxygen determines whether regions in a solid tumor will remain normoxic or display hypoxic conditions along with anaerobic metabolic processes [68, 69]. Extreme hypoxemia causes both apoptosis and necrosis to occur within necrotic areas. The hypoxic environment triggers tumor cells to reorganize their metabolism to preserve energy production together with cell survival [70].

#### 2.2.2 Hypoxia‐Inducible Factor (HIF) Activation

**FIGURE 1:**
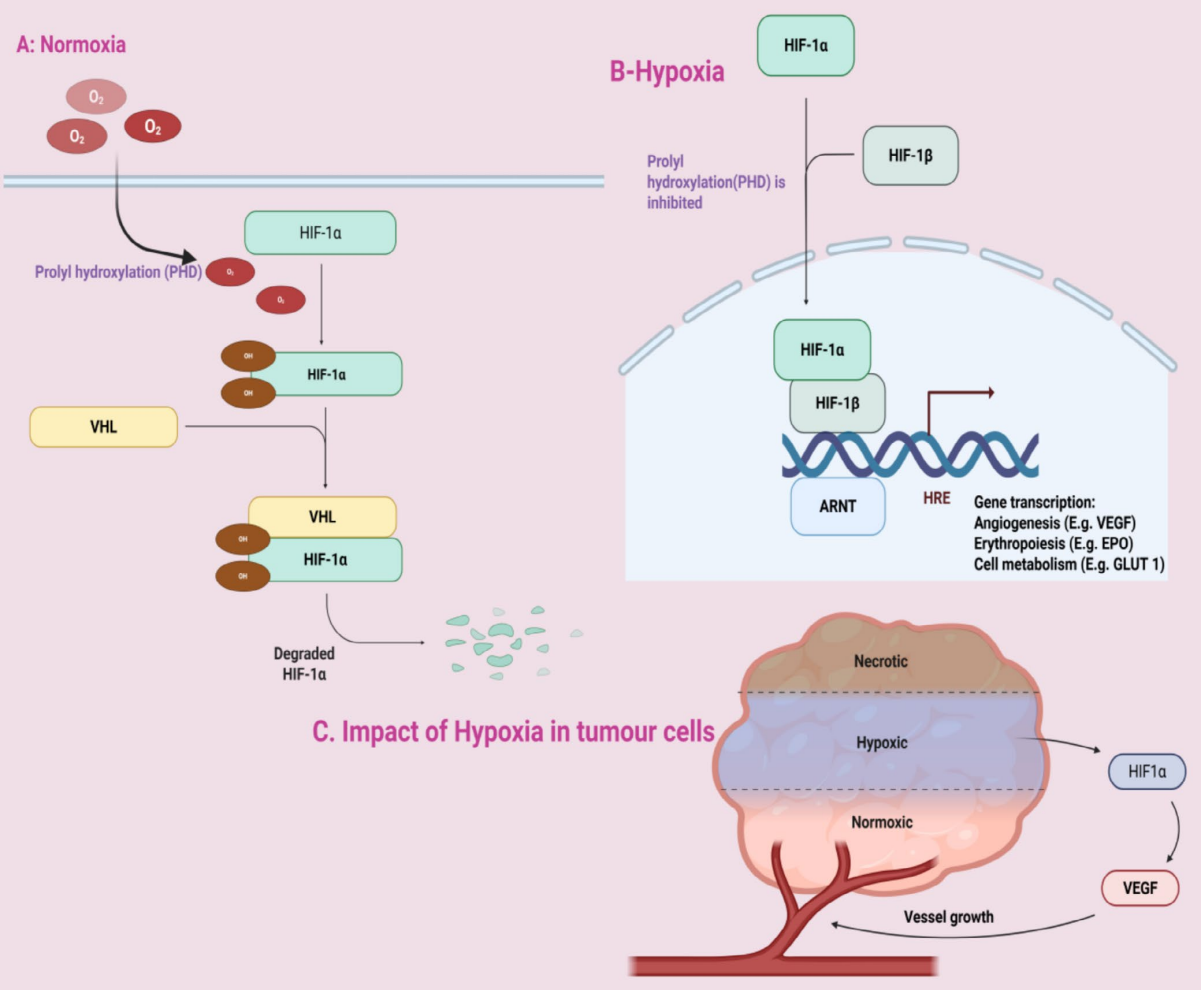
Regulation of hypoxia‐inducible factor 1 protein leading to its degradation/upregulation in varying oxygen conditions: (A) Under conditions of normal oxygen tension, or normoxia, HIF isoforms are expressed. HIFα is swiftly hydroxylated at two proline residues by a group of oxygen and α‐ketoglutarate‐dependent enzymes known as prolyl hydroxylases (PHDs). Hydroxylated HIFα is later identified by the von Hippel–Lindau (VHL) tumor suppressor protein inside the extracellular vesicle (ECV) complex. This leads to rapid polyubiquitylation and subsequent destruction by the 26S proteasome. (B) In hypoxic conditions, decreased oxygen levels in a cell result in the inhibition of PHDs. This enables HIFα isoforms to dimerize with the constitutively produced ARNT (also known as HIFβ). This transcription factor migrates to the nucleus, where it interacts with hypoxia‐responsive elements (HREs) to commence the transcription of HIF‐responsive genes. These include genes that facilitate angiogenesis, cellular metabolism, and erythropoiesis. Likewise, when VHL is mutated or absent, the ECV complex loses its capacity to identify hydroxylated HIF, leading to an elevation in HIF expression and HIF‐mediated gene transcription. (C) The impact of ARNT on HREs causes the proliferation of tumor (Created in https://BioRender.com). Modified from Robinson & Ohh [74].

The transcriptional regulators HIFs control the adaptation process to hypoxia [71]. In regular conditions, the HIF‐1α protein undergoes hydroxylation through prolyl hydroxylases, which leads to its destruction using the von Hippel–Lindau tumor suppressor mechanism [72, 73]. The hypoxic condition permits HIF‐1α stabilization to move to the nucleus, where it pairs up with HIF‐1β to activate hypoxia response elements in genes that control glycolysis and angiogenesis alongside cellular survival, leading to metabolic reprogramming in hypoxic tumor cells [60]. To adapt to hypoxia, tumor cells undergo metabolic shifts that favor survival in oxygen‐poor conditions: In brief, this process has been summarized in Figure 1. Illustrating impact in hypoxic cells, in tumor cells, and normoxic cells leading to the destruction of HIF‐1α.

#### 2.2.3 Shift From Oxidative Phosphorylation to Glycolysis (Warburg Effect)

**FIGURE 2:**
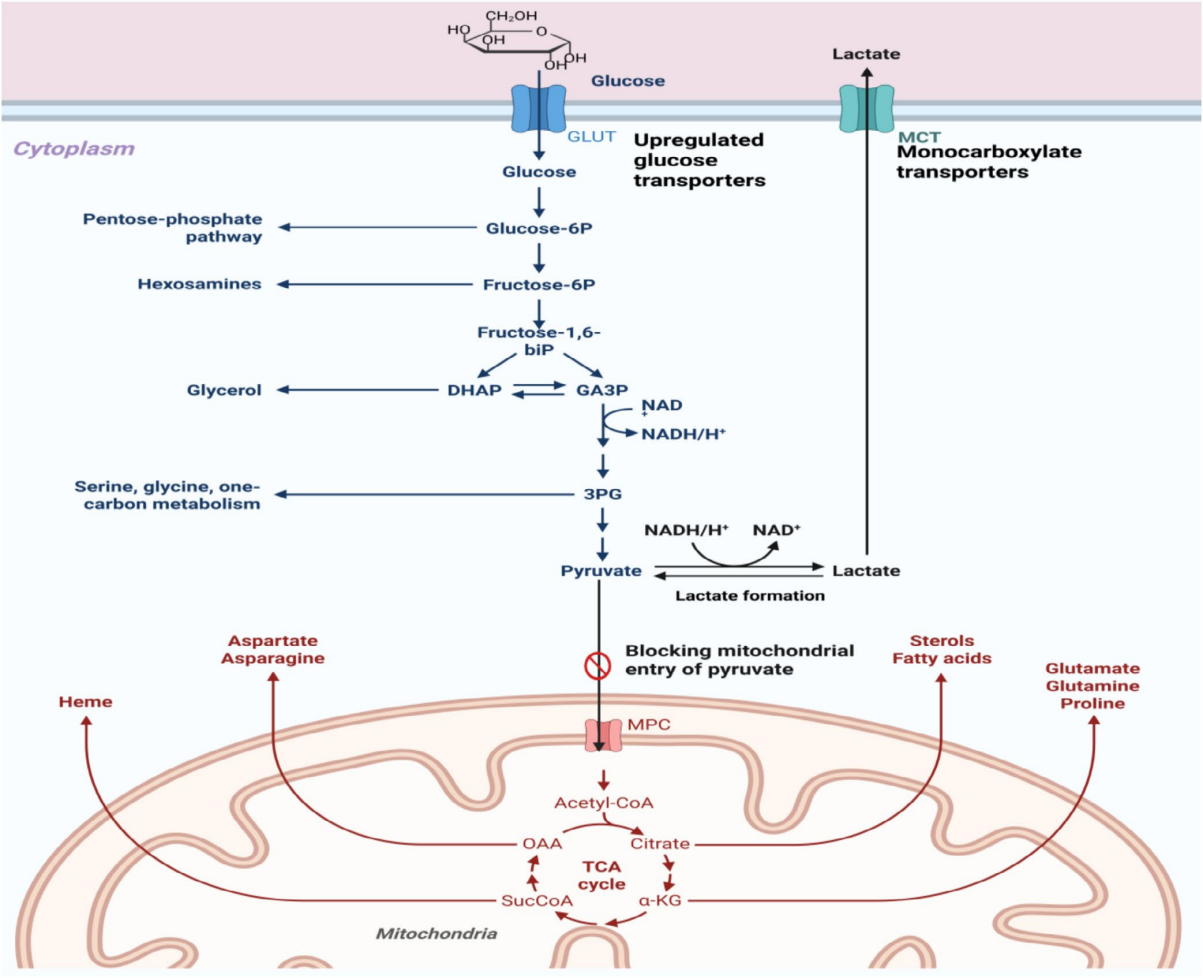
Warburg effect: (Created in https://BioRender.com). Modified from DeBerardinis, & Chandel [76].

Hypoxic tumor cells exhibit a metabolic shift toward aerobic glycolysis instead of mitochondrial OXPHOS, characterized by increased glucose import and enhanced lactate production, consistent with the Warburg effect [75]. The cellular process results from the actions of HIF‐1α which stimulates glucose transporters (GLUT) and glycolytic enzymes (upregulates GLUT1, GLUT3) and glycolytic enzymes (HK2, phosphofructokinase, LDHA), combined with Pyruvate dehydrogenase kinase 1 (PDK1) blocking mitochondrial entry of pyruvate to avert OXPHOS (Figure 2) [77, 78]. The relationship between lactate production and acidosis leads to excess lactate secretion through monocarboxylate transporters (MCT4), which causes TME acidification that increases tumor aggressiveness and suppresses immune responses while reshaping extracellular matrix (ECM) [79, 80]. Hypoxic cells rely on glutaminolysis to replenish TCA cycle intermediates and generate reducing equivalents (NADPH) for oxidative stress resistance [81]. The hypoxic environment allows cells to both replenish TCA cycle intermediates and produce NADPH which helps resist oxidative stress through glutamine metabolism. Additionally, α‐ketoglutarate enables cellular adaptability during hypoxic conditions [82, 83].

### 2.3 Mitochondrial Adaptations in Hypoxia

Under hypoxic conditions, tumor cells control the amount of oxygen that enters their mitochondria to stop ROS formation and enhance fission and mitophagy processes for eliminating damaged mitochondria while HIF‐1α stabilizes to strengthen hypoxic signaling via succinate accumulation [84, 85]. Tumors initiate blood vessel formation through pre‐existing vasculature by increasing angiogenesis because they receive insufficient oxygen during hypoxic conditions [86]. The formation of leaky and dysfunctional blood vessels through this process ensures the delivery of oxygen and nutrients for tumor progression [87]. Through HIF‐1α activation, tumors enable expression of pro‐angiogenic genes including VEGF together with angiopoietin‐2 (ANGPT2), platelet‐derived growth factor (PDGF), and matrix metalloproteinases (MMPs), which drive endothelial cell migration toward vessel formation [88, 89]. The hypoxic environment triggers disorganized and leakage‐prone blood vessels that fail to function properly, which restricts drug access and hampers immune cell entry into tumors. The main goal of antiangiogenic medications consists of vascular normalization within tumors to enhance therapeutic effectiveness.

The low oxygen environment of cancer cells causes two parallel effects: it minimizes antitumor immune responses and simultaneously enhances the presence of immunosuppressive cells [70, 90]. The programmed death‐ligand 1 (PD‐L1) protein is promoted through HIF‐α upregulation so tumor cells can disable cytotoxic T cells through PD‐1/PDL‐1 signaling pathways. The cells of myeloid origin known as myeloid‐derived suppressor cells (MDSCs) and regulatory T cells called Tregs are specifically drawn to this environment where they minimize effector T‐cell activity [91, 92]. Hypoxia reduces the ability of dendritic cells to mature along with their function to present antigens while simultaneously it weakens natural killer (NK) cell effectiveness which leads to tumor resistance against immune detection [93, 94]. The therapeutic results of immune checkpoint inhibitors become restricted by hypoxic conditions present in the TME. The challenge is to focus research on developing hypoxia‐targeting medications along with methods for oxygenation treatment and hypoxic niche reprogramming to improve immunotherapy results.

Previous research has identified hypoxia and its subsequent targets as a possible strategy to improve the efficacy of anticancer therapies. Identifying HIF‐1α has heightened interest in the modulation of hypoxia‐associated signaling pathways [95, 96]. Multiple techniques have been devised to counteract hypoxia‐induced resistance, such as radiosensitizers, hypoxia‐activated prodrugs, and HIF‐1α inhibitors [97, 98]. Certain drugs, including 2‐nitroimidazole radiosensitizers and heterocycle‐N‐oxide hypoxia cytotoxins, exhibit antiangiogenic and antimetastatic properties by obstructing HIF‐1α‐mediated signaling [99, 100]. Moreover, pharmacological suppression of HIF‐1 or its target gene PDK1 may increase tumor cell oxygen consumption, thereby augmenting the efficacy of hypoxia‐specific cytotoxins [101, 102].

## 3 Nanoparticle Engineering Strategies Targeting Vulnerabilities in Prostate Cancer

### 3.1 Nanoparticle Engineering for Targeted Delivery

Although similar engineering approaches have been applied across a range of solid tumors, this section discusses them primarily in the context of PCa biology. For example, hypoxia and tumor heterogeneity are common challenges in oncology but manifest in PCa through enhanced glycolytic reprogramming and variable PSMA expression. Modern drug delivery of drugs to target cells, tissues, and organs has undergone a transformation through nanoparticles because they provide both targeted delivery mechanisms with customized drug release profiles and enhanced delivery efficiency [43, 44, 103]. Novel nanotechnology requires engineered methodologies to choose appropriate materials and apply appropriate functionalization methods for nanoparticles to reach biological targeting destinations effectively [104].

#### 3.1.1 Types of Nanocarriers

**FIGURE 3:**
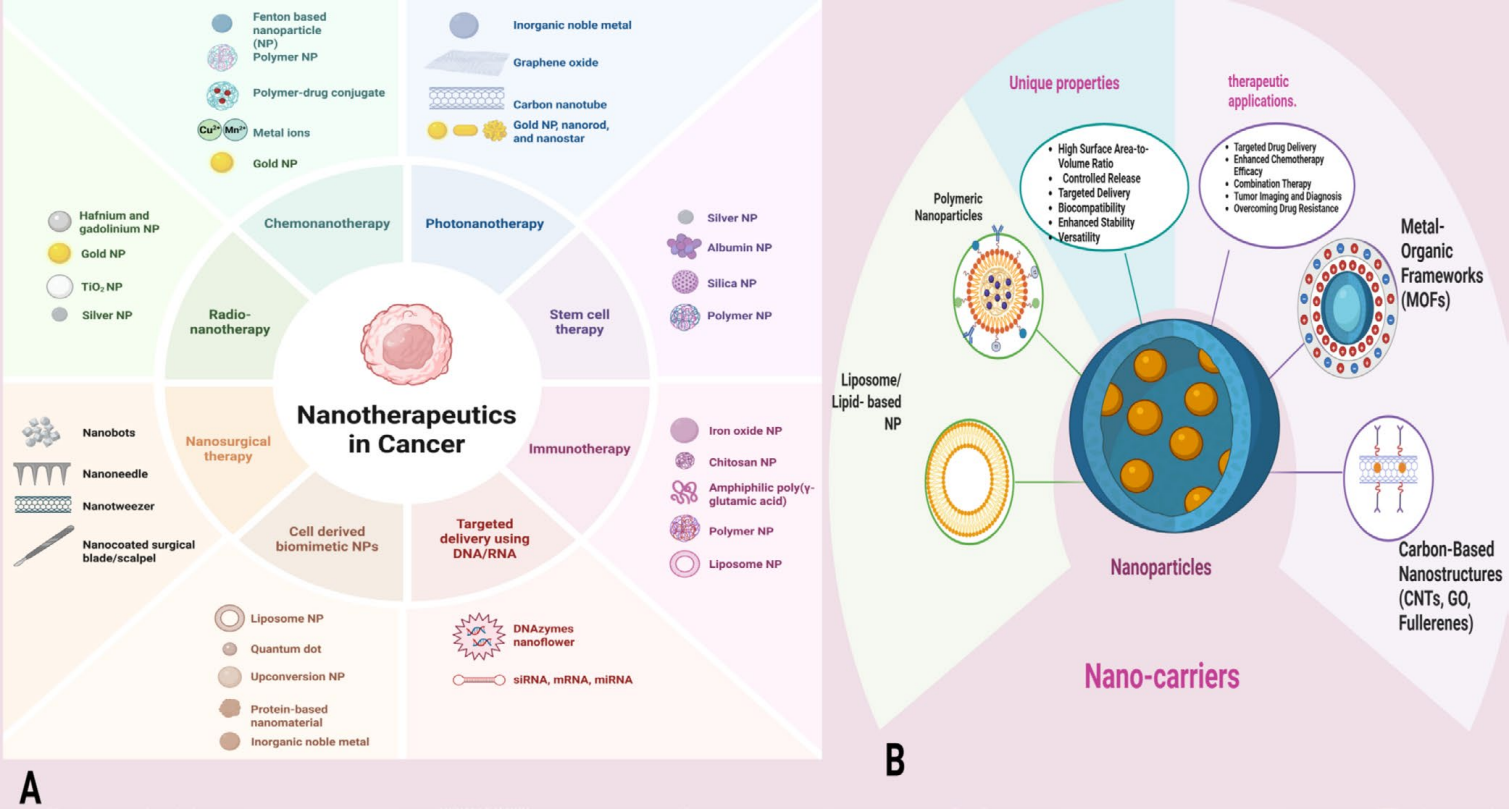
Different forms of nanotherapeutics in cancer. Adapted from Ojha et al. [105], and Uti et al. [43] (Created in https://BioRender.com), under an open access Creative Common CC BY license. (A) Classification based on therapeutic approaches. (B) Classification of nanomaterials by their intrinsic properties.

The development of nanocarriers serves to enhance the solvable properties together with stability features and precision characteristics of therapeutic agents. The main categories of nanocarriers consist of liposomes and polymeric nanoparticles (PNPs) together with metal‐based nanoparticles and other forms of nanocarriers in cancer treatment (Figure 3) [105]. The spherical vesicles named liposomes consist of phospholipid bilayers which serve to contain hydrophilic and hydrophobic therapeutic agents. These small structures provide both excellent compatibility with biological systems alongside longer blood circulation times and natural tumor tissue uptake properties [106]. Liposomal‐encased doxorubicin serves as a cancer therapy while Ambisome functions as an antifungal medication with commercial applications in Doxil for cancer therapy and Ambisome for fungal infections [107]. The delivery of drugs using PNPs demonstrates adjustable drug release rates because they incorporate biodegradable polymers such as poly(lactic‐co‐glycolic acid (PLGA)), polylactic acid (PLA), and chitosan for precise targeted drug distribution [108, 109]. Participating nanoparticles made of metal exhibit unique optical and magnetic properties for drug delivery and diagnostics and theranostics applications because of containing components like gold, silver, and iron oxide [110, 111]. The application of gold NPs exists for photothermal cancer therapy whereas iron oxide NPs serve for magnetic resonance imaging (MRI)–guided drug delivery systems [55, 112].

Previous research on nanocarrier‐based drug delivery systems has yielded substantial insights into their therapeutic efficacy, particularly for cancer therapy. Liposomes have been thoroughly investigated for their biocompatibility and capacity to encapsulate both hydrophilic and hydrophobic pharmaceuticals, enhancing bioavailability and reducing systemic toxicity [106, 113]. Nonetheless, several preceding research were constrained by challenges like early drug leakage, poor encapsulation efficiency, and inadequate targeting specificity [113]. Research on PNPs has shown potential owing to their biodegradable characteristics and adjustable drug release patterns; nonetheless, issues persist concerning repeatability in large‐scale production, stability during storage, and possible immunogenicity [108, 114]. Metal‐based nanoparticles, especially those composed of gold or iron oxide, have shown dual functionality in therapeutic and diagnostic (theranostic) applications [115, 116]. However, apprehensions over long‐term toxicity, biodistribution, and bodily elimination have impeded their practical use [115]. Moreover, whereas preclinical studies have shown improved tumor penetration and retention via the enhanced permeability and retention (EPR) effect, human trials have not consistently reproduced these results, indicating a translational gap in effectiveness [117]. Additionally, the intricate TME, heterogeneity across patients, and the possibility of nanoparticle agglomeration provide extra constraints [118]. Knowledge deficiencies remain about the prolonged safety of diverse nanocarriers, their interactions with immune cells, and the processes governing their transport across biological barriers. There is a need for more extensive comparison studies that assess various nanocarrier systems under standardized circumstances to determine the most suitable platforms for certain therapeutic applications. Subsequent investigations need to concentrate on the incorporation of sophisticated targeting ligands, responsive release mechanisms, and real‐time imaging functionalities to enhance specificity and therapeutic efficacy. It is essential to address these constraints and information gaps to fully exploit the promise of nanocarrier‐based drugs delivery in cancer and other disease conditions.

#### 3.1.2 Chemically Stimulated Hypoxia‐Responsive Nanomaterials

Hypoxia, a defining trait of several solid tumors and ischemic tissues, creates a distinctive biochemical milieu that has been progressively used for targeted treatment strategies [119]. Although most hypoxia‐responsive nanomedicines primarily target the modulation of the HIF‐1α signaling pathway, a unique and potent category of nanomaterials depends on chemical stimulation mechanisms. These chemically sensitive systems use functional moieties that experience bioreductive or redox‐triggered changes under low oxygen circumstances [98, 120, 121]. These transformations include linker breakage, alterations in polarity or solubility, bond rearrangements, and enzyme‐mediated reductions, all leading to the regulated release or activation of medicinal substances. Significantly, these techniques operate independently of varying HIF‐1α expression across various tumor types, providing a more broadly applicable methodology [117, 122]. Essential chemical groups used in these systems include nitroimidazoles, azo linkages, quinones, sulfonamides, and other redox‐sensitive linkers. These may be designed as nanocarriers, including liposomes, micelles, dendrimers, and gold nanoparticles, to attain increased specificity, bioavailability, and reduced systemic toxicity [123, 124].

Prominent examples are nitroimidazole‐functionalized liposomes that experience enzymatic reduction under hypoxic conditions to discharge their cargo, and azo‐linked polymeric micelles that disintegrate upon cleavage by azoreductases in low oxygen settings [121]. Quinone derivatives, including naphthoquinones, undergo reduction to hydroquinones in hypoxic environments, triggering chain reactions that either release pharmaceuticals or alter nanoparticle architecture. Sulfonamide and N‐oxide‐based linkers have been integrated into intelligent nanocarriers for targeted medication delivery [125] (Table S1). These platforms not only improve the effectiveness of chemotherapeutic drugs but also diminish off‐target effects. Moreover, researchers are beginning the incorporation of these chemical moieties into multimodal platforms for theranostics, merging medicinal administration with real‐time imaging [126, 127]. Notwithstanding these gains, obstacles persist, including intricate synthesis pathways, possible off‐target activation in tissues with variable oxygen levels, and a paucity of translational research. Future research is anticipated to concentrate on hybrid nanomaterials that integrate biological and chemical response, including artificial intelligence (AI)–assisted design for optimization. Chemically triggered hypoxia‐responsive nanomaterials together signify a significant advancement in the development of next‐generation precision therapies for cancer and other hypoxia‐related disorders.

#### 3.1.3 Surface Modifications for HIF‐1α Targeting

The master regulation molecule HIF‐1α controls cell adjustment to hypoxic situations and guides tumor development together with blood vessel growth and forces tumors to become resistant to therapy [128]. Treatment efficacy can improve when engineered nanoparticles are focused on HIF‐1α because such nanoparticles can specifically block hypoxic tumor cells. The methods for improving specificity and targeting efficiency involve antibody functionalization and peptide ligands and aptamers alongside hypoxia‐responsive coatings [55, 129]. Functionalizing the surface of nanoparticles with targeting moieties significantly improves their specificity and delivery efficiency. For instance, conjugation with monoclonal antibodies enables selective recognition of target cell receptors, while peptide ligands facilitate more efficient accumulation at the desired site. Similarly, short single‐stranded DNA or RNA molecules known as aptamers can be engineered to bind with high affinity and specificity to HIF, thereby enhancing targeted therapeutic action [130]. Hypoxia‐responsive coatings enable controlled drug release only when the exposure environment becomes hypoxic and HIF‐1α reaches high expression levels [19, 20]. Hypoxic tumor targeting along with resistant‐to‐drug therapies strengthens through the utilization of these specific delivery systems.

Experts have tested several strategies involving nanoparticles aimed at targeting HIF‐1α as a way to manage the hypoxia‐caused resistance of tumors to treatment [131]. Useful results have been observed with antibody‐functionalized nanoparticles and materials that respond to hypoxia, including greater drug concentration in deficient regions, improved tumor regression, and less harmful impact on healthy cells [132]. The strong and special binding of aptamers to HIF‐1α makes it easier to target the protein [133]. However, there are still certain restrictions to deal with. Many approaches revealed that they do not effectively penetrate solid tumors and have inconsistent release of drugs [133]. Both antibody and peptide technologies can lead to an immune response, and the use of aptamers in vivo may cause them to break down [134]. Additionally, scalability and reproducibility of nanoparticle formulations pose challenges for clinical translation. Despite promising laboratory findings, only a small amount of research has gone farther than initial trials. Important knowledge gaps involve the long‐term outcomes of nanoparticle treatments, how they are removed from the body, and how diverse tumors respond to them. Resolving these deficiencies is necessary in the treatment of PCa, targeting HIF‐1α.

### 3.2 Mechanisms of Action

**FIGURE 4:**
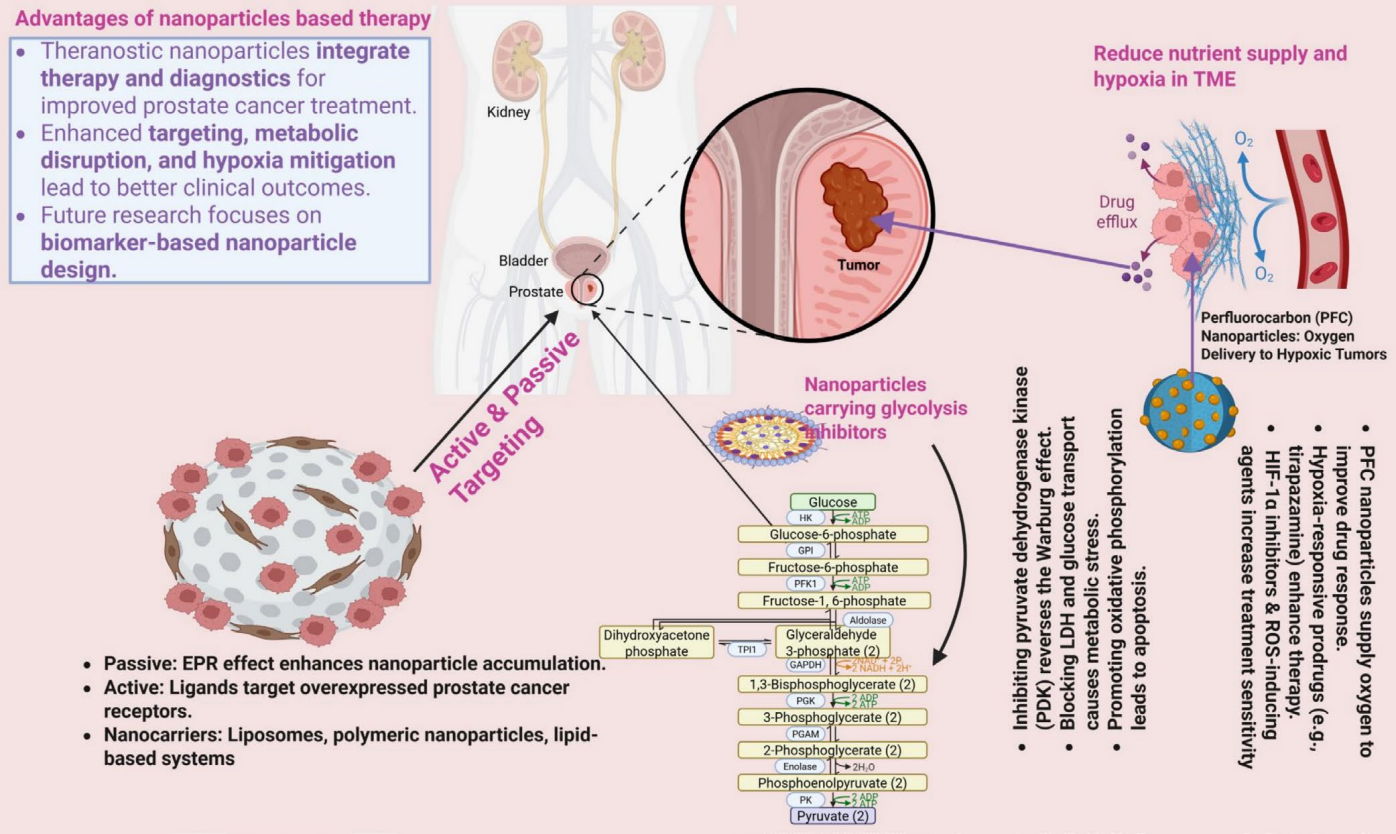
Theranostic nanoparticles for prostate cancer therapy: Mechanisms of action (Created in https://BioRender.com).

Drugs contained in TNPs utilize three pathways that achieve drug delivery to tumors through active targeting, passive targeting and through glycolysis inhibition to disrupt tumor metabolism (Figure 4). These nanoparticles also work to defeat drug resistance triggered by tumor hypoxia.

#### 3.2.1 Active and Passive Targeting of Prostate Tumors

Specific PCa treatment by nanoparticles combines passive and active targeting elements to deliver more medication to tumors yet maintain low systemic toxic effects [135]. Nanoparticles utilize the EPR effect to accumulate more frequently within tumorous tissue environments. Drugs and imaging agents together with chemotherapeutic agents are delivered more effectively through the utilization of liposomes along with PNPs and lipid‐based nanosystems [117, 136]. Nanoparticles succeed in active targeting strategies through the attachment of specific ligands that bind with receptors that are overexpressed on PCa cells while using PSMA ligands, folate receptors, and antibodies or peptides [55, 117]. The precise delivery approaches provide better drug availability together with decreased unintended side effects and superior clinical results.

#### 3.2.2 Disrupting Glycolytic Pathways Using Nanoparticles

PCa cells consume aerobic glycolysis as their primary energy‐producing mechanism under conditions that contain available oxygen [137]. Nanoparticles function as disruptive agents against glycolysis, which helps stop tumor advancement. The interaction between glycolytic inhibitors such as 2‐deoxy‐D‐glucose (2‐DG) and GLUTs warrants careful consideration. Since 2‐DG enters cells via GLUTs, inhibition of these transporters can inadvertently reduce 2‐DG uptake and consequently diminish its glycolytic inhibitory effects. Therefore, therapeutic strategies targeting both glycolysis and glucose transport must account for this competition either by optimizing dosing regimens or employing sequential inhibition approaches to maximize therapeutic efficacy. Administrating glycolytic inhibitor 2‐DG through nanoparticles improves its effectiveness and makes it selectively target cancer cells [138]. Nanoparticles support mitochondrial OXPHOS by blocking the activity of PDK, thus promoting both Warburg effect reversal and apoptotic cell death. Nanoparticles have a detrimental effect on LDH inhibitors, which prevents glycolysis maintenance, thus causing metabolic distress that leads to cancer cell mortality [19, 139]. Cancer cells lose their energy supply, and the therapy effectiveness improves when nanoparticles with GLUT inhibitors prevent glucose transport through cell membranes [19, 140]. The targeted delivery of nanoparticles enables specific reduction of gene expression that affects glycolysis, which leads to tumor inhibition by disrupting cellular metabolism.

#### 3.2.3 Overcoming Drug Resistance in Hypoxic Tumors

Drug resistance alongside cancer progression becomes more likely when prostate tumors create hypoxic regions because of their rapid expansion beyond available blood supply. Perfluorocarbon (PFC) nanoparticles attract attention as an anti‐hypoxia solution because these nanoparticles make oxygen available at tumors to reduce treatment challenges [141, 142]. The delivery of hypoxia‐sensitive drugs becomes possible by encapsulating hypoxia‐responsive prodrugs such as tirapazamine [19]. The anti‐hypoxia therapies of nanoparticles include HIF‐1α inhibitors along with antiangiogenic agents combined with immune checkpoint inhibitors [143, 144]. Treatment response and immune surveillance restoration occur in hypoxic tumors by using these nanoparticles. When utilized to enhance oxidative stress in cancer cells, ROS‐inducing agents make tumors more sensitive to radiotherapy and chemotherapy [145].

TNPs represent an advanced method for PCa treatment since they unite diagnostic imaging with therapeutic delivery [146]. These nanoparticles implement three key mechanisms of action through drug targeting mechanisms and their ability to hinder glycolysis while overcoming drug resistance caused by hypoxia to deliver superior results than typical treatments. The clinical transformation of advanced therapeutic nanoparticles will benefit from current biomedical developments related to biomarker identification and particle design.

### 3.3 Theranostic Nanoparticles: Monitoring Tumor Hypoxia and Controlled Drug Release for Better Cancer Treatment

#### 3.3.1 Dual Functionality of TNPs: Imaging and Therapy

TNPs consist of diagnostic imaging components together with therapeutic agents that enable both diagnostic evaluation and treatment processes [147]. Various nanomaterials including metallic nanoparticles, as well as PNPs, lipid‐based nanoparticles, quantum dots, and carbon‐based nanomaterials serve in their engineering process [148] The combined functionality of these nanoscale substances enables MRI‐based, PET‐based, CT‐based, fluorescence, optical imaging, and photoacoustic imaging of tumors in real time. Therapeutic nanoparticles generate time‐sensitive information about drug movement across the body while showcasing tumor developments and medication treatment indications [148] The drug delivery function of TNPs acts as a protective carrier system for medications that improves the amount of drug available in the body and boosts dissolution properties while guiding specimens precisely toward tumors through stimulus‐dependent drug release mechanisms [1, 149]. Technology enables the real‐time monitoring of drug distribution together with tumor growth and treatment effects.

#### 3.3.2 Real‐Time Monitoring of Tumor Hypoxia

Aggressive solid tumors commonly show tumor hypoxia because of their excessive growth outrunning their blood supply capability. Hypoxic conditions induce drug resistance along with poor prognosis and increased metastasis and lead to activation of HIF‐1α, which stimulate survival pathways as well as activate angiogenesis [150]. Tumor hypoxia detection and imaging become possible through the utilization of three separate techniques: oxygen‐sensitive nanoprobes, hypoxia‐responsive MRI contrast agents and HIF‐1α‐targeted nanoprobes [151]. The payload of these nanoparticles releases specifically in hypoxic conditions through three main strategies which include pH‐sensitive systems and redox‐responsive systems along with enzyme‐activated systems that use hypoxia‐expressed enzymes [152]. The designed strategies deliver anticancer drugs precisely to the tumor location which lowers systemic harm while improving treatment effectiveness.

#### 3.3.3 Controlled Drug Release and Enhanced Efficacy

TNPs use different stimulus‐sensitive methods to control drug release through such elements as pH‐sensitive release, temperature‐sensitive release, magnetic field‐controlled release, and light‐responsive release [153]. The nanoparticles operate through tumor‐targeting capabilities followed by magnetic control of their release and near‐infrared light activation in targeted tissues [154]. The application of these nanoparticles as drug carriers leads to enhanced therapeutic results because they minimize drug‐related unwanted effects along with maintaining drug integrity in blood and achieving detailed drug tracking during real‐time observations. The combination of chemotherapy drugs with gold nanoparticles leads to increased accumulation in tumors while simultaneously minimizing cardiac damage based on preclinical research findings [155, 156].

#### 3.3.4 Applications of TNPs in Cancer Therapy

Nanoparticles in theranostic applications represent the new frontier in cancer nanomedicine since they link imaging with therapeutic functions within a unified platform [156]. The platform provides customizable treatment plans which rely upon tumor feedback as well as nanomedicine method choices that match individual patients and provides simultaneous drug and light‐based therapeutic methods [157]. These systems manage drug resistance by utilizing two methods which involve avoiding multidrug resistance systems then silencing resistance‐associated genetic material. Researchers currently focus on creating eco‐friendly nanomaterials that reduce health risks during medical applications as well as implementing intelligent analysis systems that process theranostic data in real‐time and establishing multifunctional nanoparticles capable of treating different cancer types simultaneously [158]. Research and development of TNPs encounters obstacles from regulatory restrictions and compatibility difficulties with human tissue while requiring improvements to manufacturing scale and remaining limited by high design expenses [159].

## 4 Targeting HIF‐1α and Downstream Metabolites With Theranostic Nanoparticles

HIF‐1α overexpression creates aggressive tumor phenotypes so researchers consider it a vital therapeutic target in cancer management [160]. The combination approach of therapeutic and diagnostic features known as TNPs shows potential for targeted HIF‐1α inhibition. Nanotechnology‐based drug systems possess the ability to deliver precise HIF‐1α inhibitors while simultaneously improving the NP uptake by cells, drug bioavailability, and enabling continuous patient treatment evaluation without creating systemic harm [55, 161]. The development of nanoparticles for use in research includes liposomes together with PNPs, dendrimers, inorganic nanoparticles, and lipid‐based nanocarriers.

### 4.1 HIF‐1α Inhibition Strategies

Two Forms of HIF‐1α Inhibition Exist Directly and Indirectly Through Targeting Methods [162, 163].

#### 4.1.1 Direct HIF‐1α Inhibitors

Stopping HIF‐1α operates through three distinct methods: decreasing its expression level, obstructing its welding union, and blocking its actions as a transcription factor. Blockade of HIF‐1α transcriptional activity, destabilization of its protein structure, and disruption of its protein–protein interactions can be achieved using small‐molecule inhibitors. For example, PX‐478 suppresses HIF‐1α expression by inhibiting its mRNA translation and promoting proteasomal degradation, ultimately reducing HIF‐1α–mediated gene transcription. YC‐1, on the other hand, enhances HIF‐1α degradation by stimulating prolyl hydroxylase activity and inhibiting HIF‐1α nuclear accumulation, thereby impairing its transcriptional activity. Another small molecule, Acriflavine, disrupts the dimerization of HIF‐1α with HIF‐1β, preventing formation of the active HIF complex [164].

The inhibition of HIF‐1α through small interfering RNA (siRNA) as part of RNA interference (RNAi) represents a direct method to knock down its messenger RNA (mRNA) [165, 166]. The clinical use of siRNA remains limited due to three essential problems which include stability issues alongside off‐target effects and delivery difficulties [167]. TNPs resolve major delivery barriers by providing siRNA stabilization in combination with targeted delivery mechanics and by enabling dynamic assessment of siRNA performance. Laboratory studies have demonstrated that lipid nanoparticles containing HIF‐1α siRNA resulted in major tumor reduction [168]. Multiple imaging and RNA interference therapy functions can be achieved by using iron oxide‐based nanoparticles.

#### 4.1.2 Indirect Targeting via Upstream Regulators

Medical practitioners can manage HIF‐1α protein at critical stages through active modulation of its initial stimulatory factors. The action of Prolyl Hydroxylase (PHD) triggers HIF‐1α stabilization in conditions when normal cells begin the process of HIF‐1α degradation by hydroxylation [169, 170]. When PHD activity declines in hypoxic conditions, it results in increased levels of HIF‐1α. Alpha‐ketoglutarate analogs and iron oxide nanoparticles serve as PHD restorative molecules which nanoparticles can deliver to cells. Inhibition of the PI3K/Akt/mTOR pathway, a major upstream regulator of HIF‐1α, leads to reduced HIF‐1α translation [171]. Nanoparticles that contain mTOR inhibitors with PI3K inhibitors effectively decrease levels of HIF‐1α protein. HIF‐1α downregulation occurs because ROS (ROS) serve as regulators of this degradation [172]. TNPs that release ROS‐inducing agents create oxidative stress conditions to achieve this goal. Hospital personnel can employ bortezomib proteasome activators to selectively degrade HIF‐1α while maintaining live monitoring of treatment outcomes. Treatment methods exist to enhance both the stability and activity levels of HIF‐1α during cancer interventions [173].

HIF‐1α with its downstream metabolites can be targeted effectively through TNPs as a new approach for cancer therapy. The therapeutic benefits from HIF‐1α targeting will emerge from direct inhibition through small molecules or siRNA, while indirect targeting uses PHD, PI3K/Akt/mTOR, ROS, and proteasomal degradation pathways. Medical researchers should focus on creating biocompatible nanoparticles that recognize tumors because this advancement could establish better HIF‐1α‐targeted therapy with better patient results and treatment success.

### 4.2 Theranostic Nanoparticles for Glycolysis Disruption

The therapeutic advances use three distinct methodologies such as enzyme function regulation through tumor‐specific ligand binding and nanoparticle reaction response elements and enzyme‐specific modulation [174]. TNPs possess design features to block enzymes that lead to impaired glycolytic flux which ultimately suppresses tumor growth. Since blocking glycolysis makes tumor cells more receptive to chemotherapeutic drugs, doxorubicin, paclitaxel, and cisplatin, researchers have discovered that this occurs because glycolysis inhibition disrupts cellular energy supply lines and weakens drug resistance patterns. Nanoparticles which carry glycolysis‐inhibiting agents together with chemotherapy drugs enable long‐term drug release and enhance combined effects that eliminate cancer cells while limiting systemic side effects [141]. Targeting peptides during functionalization enable drug delivery systems to find tumors more efficiently, which leads to improved therapy effects by reducing unintended side effects [141].

The effect of radiotherapy can become stronger when researchers stop glycolysis because this prevents DNA repair through ATP and improves DNA damage from radiation. Radiotherapy effects intensify through the combination of TNPs with glycolysis inhibition agents and radiosensitization compounds to enhance the damage of DNA and increase oxidative stress. Tumors treated with PFC‐based systems that deliver oxygen gain better sensitivity to radiation due to the reversal of hypoxic conditions [175]. Various limits persist in nanotherapeutic applications such as biological safety and toxicity effects, and difficulties with tumor heterogeneity together with the complex path to clinical application and regulatory barriers and challenges merging multiple treatment modalities [141]. The safety of nanomaterials into the future demands complete toxicological evaluations since these must be established for clinical purposes. Surface modification using biocompatible polymers prolongs the circulation time of nanoparticles and decreases their entry into the reticuloendothelial system [117].

The metabolic patterns of tumors show heterogeneity across different cancer types together with their individual regions that warrant specific therapeutic methods for maximum effectiveness [176]. The creation of multitasking nanoparticles designed to address multiple tumor metabolic workings in parallel may prove effective against cancer metabolic flexibility. Clinical implementation of nanoparticles along with regulatory requirements demands consistent methods for making nanoparticles and testing them for reliability while demonstrating safety and effectiveness according to medical regulations. The therapeutic combination of angiogenesis inhibitors with various drugs disrupts tumor support networks of both metabolic pathways and vascular pathways leading to improved patient outcomes [177]. TNPs provide cancer cell treatment and imaging capabilities through their ability to block glycolysis functions. These nanosystems demonstrate excellent potential to advance cancer therapy since their delivery of HIF‐1α inhibitors in combination with glycolytic enzyme control and chemotherapy treatment and radiotherapy administration enables better outcomes.

### 4.3 Nanoparticle‐Based Drug Combinations

Nanoparticle‐mediated medication combinations provide a potential approach to augmenting cancer treatment by the simultaneous administration of metabolic inhibitors and immunomodulators [178]. These sophisticated delivery methods augment medication bioavailability, reduce systemic toxicity, and boost therapeutic effectiveness. The action mechanism of these combinations involves 2‐DG, which competitively inhibits glycolysis by targeting hexokinase, thereby depriving cancer cells of vital energy sources [179]. Nonetheless, its clinical use has been impeded by systemic toxicity and inadequate selectivity. The use of nanoparticles for the delivery of 2‐DG improves its targeting of tumors and diminishes off‐target effects [180].

LDHA inhibitors, like FX11 and oxamate, have potential in preclinical models; nevertheless, their restricted solubility and bioavailability need nanoparticle‐mediated delivery approaches. The benefits of nanoparticle‐mediated co‐delivery of metabolic inhibitors include improved tumor targeting, synergistic therapeutic effects, regulated release and extended circulation, as well as the mitigation of drug resistance [181]. Instances of nanoparticle‐based metabolic inhibitor combinations include liposomes containing 2‐DG and LDHA inhibitors, PNPs encapsulating metabolic inhibitors, and functionalized metallic nanoparticles coupled with metabolic inhibitors.

Furthermore, nanoparticle‐based immunomodulatory strategies have surfaced as a possible method to enhance therapy effectiveness [182]. These techniques include checkpoint blocking treatment, cytokine administration, and metabolic reprogramming of immune cells [183]. Nanoparticles enhance the administration of immune checkpoint inhibitors, mitigate systemic toxicity, and augment effectiveness. They may also facilitate the repolarization of tumor‐associated macrophages into an antitumorigenic M1 phenotype. Instances of nanoparticle‐mediated immunomodulatory strategies include polymeric micelles for checkpoint inhibition, liposomal formulations for interleukin‐2 administration, and iron oxide nanoparticles for macrophage polarization [184].

In sum, nanoparticle‐based medication combinations provide a potential approach to augmenting cancer treatment via the simultaneous administration of metabolic inhibitors and immune‐modulators. These sophisticated delivery methods augment medication bioavailability, reduce systemic toxicity, and boost therapeutic effectiveness. Future research must concentrate on refining nanoparticle formulations to attain accurate targeting, regulated medication release, and diminished resistance mechanisms. The amalgamation of nanotechnology with metabolic and immune‐targeted medicines has significant promise to transform cancer treatment methodologies.

### 4.4 Nanoparticle‐Enabled Delivery of Ferroptosis Inducers

Ferroptosis, a regulated form of cell death driven by iron‐dependent lipid peroxidation, has recently emerged as a promising therapeutic vulnerability in PCa [185]. The hypoxic TME promotes metabolic plasticity and therapy resistance, but it also sensitizes malignant cells to ferroptotic stress. Recent studies highlight mechanistic links between ferroptosis and noncoding RNAs (ncRNAs). For instance, an enhancer variant was shown to regulate nuclear receptor coactivator 4 (NCOA4)–driven ferritinophagy and ferroptosis, thereby modulating iron release and lipid peroxidation sensitivity in PCa [186]. Likewise, the enhancer RNA long noncoding RNA promoting transferrin (LTFe) was found to induce ferroptosis via transcriptional activation of lactotransferrin (LTF), underscoring the role of ncRNAs as ferroptosis regulators [187, 188, 189, 190]. These findings suggest that ncRNA‐mediated ferroptosis pathways can be exploited as therapeutic targets.

Nanoparticle‐enabled delivery provides a powerful approach to translate these insights into therapy. Conventional ferroptosis inducers, such as erastin or RAS‐selective lethal 3 (RSL3), face limitations including poor solubility, rapid clearance, and systemic toxicity [191]. Nanoparticle platforms such as lipid‐based carriers, polymeric micelles, and iron oxide nanostructures can overcome these barriers by enhancing drug solubility, prolonging circulation time, and enabling tumor‐specific accumulation [43]. Importantly, nanoparticles can be engineered to co‐deliver ferroptosis inducers alongside ncRNA modulators, thus directly targeting the AR ferroptosis crosstalk implicated in PCa progression and therapy resistance. For example, encapsulating erastin within lipid nanoparticles functionalized for PSMA targeting could improve selective delivery to prostate tumors while minimizing systemic toxicity [192]. Similarly, co‐delivery of ferroptosis inducers with small interfering RNAs (siRNAs) or antisense oligonucleotides against ferroptosis‐suppressive ncRNAs could amplify tumor‐specific lethality [193]. Nanoparticle‐mediated delivery of ferroptosis inducers represents a frontier in PCa therapy. By integrating ferroptosis modulators, ncRNA regulators, and precision nanotechnology, this strategy holds potential to overcome hypoxia‐driven resistance and broaden the therapeutic landscape for advanced PCa.

### 4.5 Next‐Generation Functionalization and Clinical Translation Barriers of Nanoparticles

While PSMA‐targeted nanoparticles have demonstrated remarkable specificity and therapeutic potential in PCa, further advances in nanoparticle engineering are required to address persistent biological and translational limitations. Recent studies highlight the promise of biomimetic coatings, such as exosome‐mimicking nanoparticles, which leverage the body's endogenous communication systems to enhance biocompatibility and immune evasion [194, 195]. Similarly, surface functionalization with erythrocyte or leukocyte membranes has been shown to reduce clearance by the reticuloendothelial system and prolong systemic circulation, thereby improving tumor accumulation and therapeutic efficacy [196, 197].

Another emerging strategy involves immune evasion through surface modification. Functionalization with proteins such as CD47, which delivers a “do not eat me” signal to macrophages, has been reported to significantly decrease phagocytosis and extend nanoparticle half‐life [198]. These approaches are particularly relevant for PCa, where repeated dosing may otherwise lead to immune sensitization and reduced therapeutic benefit.

Beyond biological coatings, AI–driven nanoparticle design has gained momentum as a next‐generation approach. Machine learning (ML) algorithms can predict optimal particle size, surface charge, and ligand density, allowing rational design of formulations with improved pharmacokinetic profiles and therapeutic outcomes [199, 200, 201]. By integrating AI into nanoparticle optimization, it may become possible to tailor delivery systems for patient‐specific tumor heterogeneity in PCa.

Despite these innovations, translation to clinical practice remains challenging. Manufacturing complexity, batch‐to‐batch variability, and scalability of biomimetic coatings present significant barriers to regulatory approval. In addition, immunogenicity concerns remain, particularly when employing exosomal or cell membrane‐derived materials. Regulatory frameworks are still evolving to accommodate hybrid and AI‐designed nanomedicines, requiring extensive safety and efficacy validation. Importantly, experiences from ongoing clinical trials in PCa, such as those evaluating PSMA‐targeted docetaxel nanoparticles (e.g., BIND‐014), have highlighted practical limitations including limited patient stratification, unexpected immune responses, and production scalability [14].

In sum, next‐generation functionalization strategies including biomimetic coatings, immune evasion mechanisms, and AI‐driven design offer exciting opportunities to improve nanoparticle performance in PCa therapy. However, their successful clinical implementation will require parallel progress in addressing immunogenicity, manufacturing, and regulatory hurdles. Bridging these gaps is essential to move nanoparticle‐enabled precision oncology from preclinical innovation toward meaningful clinical impact.

#### 4.5.1 Combination Nanomedicine Strategies in PCa

While previous parts of this review illustrate the promise of implementing tumor‐targeting nanoparticles (TNPs) for tumor targeting in combination with systemic therapies, a dedicated synthesis defines the mechanistic synergies and translational aspects that are most likely to be achieved. Herein we describe rational combinations of TNPs with immunotherapy, poly(ADP‐ribose) polymerase (PARP) inhibitors, and ADT/AR pathway inhibitors with special emphasis on principles of design, sequencing, and translation for PCa.

##### 4.5.1.1 Tumor‐Targeting Nanoparticles with Immunotherapy

TNPs are capable of (i) delivering neoantigens and adjuvants to lymph nodes for T‐cell primings; (ii) modifying the immunosuppressive TME as a result of localized delivery of immunostimulin (stimulator of interferon genes (STING) or toll‐like receptor (TLR) agonists); and (iii) co‐delivery of cytotoxic and/or radiosensitizing payloads to promote immunogenic cell death and improve responses to immune checkpoint blocking agents. Prostate cancer antigens for high intratumoral exposure and underexposure due to immunologic adverse response can include PSMA‐targeted or other prostate‐homing ligands [202].

##### 4.5.1.2 Tumor‐Targeting Nanoparticles Carried With Poly(ADP‐Ribose) Polymerase (PARP) Inhibitors

Given the subsets of PCa harboring homologous recombination repair defects, TNPs could be employed for the purposes of synthetic lethality by co‐encapsulating PARP inhibitors with DNA damage‐inducing agents or Ataxia telangiectasia and Rad3‐related (ATR)/checkpoint kinase 1 (CHK1) inhibitors. Nucleic acid cargoes (siRNA/CRISPR) encapsulated in TNPs are able to temporarily disarm the repair mechanisms and thus extend the sensitivity in PARP inhibition [203].

##### 4.5.1.3 Tumor‐Targeting Nanoparticles With Androgen Deprivation Therapy (ADT) and Androgen Receptor (AR) Pathway Inhibitors

Nanocarriers provide tumor‐specific concentration of anti‐androgens, AR degraders, or AR‐axis siRNAs and allow triplet combinations (e.g., ADT + TNP loaded with taxane + immunomodulator). Sequential hypotheses of AR blockade to decrease immune exclusion are followed by TNP‐mediated immune lysine and antigen release [204].

### 4.6 Clinical and Preclinical Advances

Current global health challenges require further development of progressive diagnostic and treatment strategies for PCa. Nanoparticles that provide both diagnostic and therapeutic functionalities have become a strategic method to enhance precise cancer medicine in oncologic care. The latest clinical and preclinical developments concerning TNPs for PCa treatment are presented in Table S2. This table summarizes key research outcomes from both live animal and test tube experiments while evaluating their success in animal testing and identifying main issues related to biocompatibility as well as toxicity and drug resistance along with tumor heterogeneity. The table reviews the existing clinical trial status as well as personalization possibilities in nanomedicine and prospective research pathways for the field. The summary can help researchers optimize PCa nanoparticle‐based theranostics through its identification of challenges and suggested future directions.

Besides the data in Table S2, other essential discoveries shed more light on how TNPs work in PCa. Abraxane (albumin‐bound paclitaxel) demonstrates that similar treatment approaches based on nanoparticles are effective against PCa, though not prostate‐specific [205, 206]. In addition, prostate tumors are detected more precisely by nanoparticles that target PSMA, and initial preclinical clinical studies suggest they might be useful in both scanning and treating the disease [207]. Preclinical animals treated with a liposomal docetaxel formulation targeted by PSMA have better absorption and more effective cytotoxic agents [208, 209]. Moreover, coupling gold nanoparticles (AuNPs) with imaging agents has been used to improve photoacoustic imaging accuracy [210, 211]. Although it is promising, this therapy still deals with issues such as immune system clearance, unintended toxicity, and patient differences. It is becoming necessary to synergize nanomedicine with genomics and AI to develop cancer treatments that are better suited to each person and their cancer targets.

## 5 Translational Challenges and Opportunities: Preclinical Promise vs. Clinical Reality

To strengthen the translational relevance of nanoparticle‐based strategies in PCa and solid tumors, it is critical to critically appraise how preclinical efficacy compares with clinical outcomes. While rodent models consistently demonstrate robust nanoparticle accumulation through the EPR effect, clinical translation has been far less consistent [212]. Factors such as tumor heterogeneity, stromal barriers, vascular normalization, and patient‐specific variability limit reproducibility in humans [213]. Several nanoparticle formulations that showed strong preclinical antitumor activity have failed to meet efficacy endpoints in phase II/III trials, highlighting the disconnect between mechanistic promise and therapeutic reality [214, 215, 216].

A major limitation in PCa nanomedicine lies in the disconnect between preclinical promise and clinical reality. For example, PSMA‐targeted systems such as BIND‐014 achieved strong tumor regression in xenografts yet delivered only modest outcomes in phase II trials, largely due to heterogeneous PSMA expression across patients [217]. Similarly, liposomal docetaxel and albumin‐bound paclitaxel showed safety and improved pharmacokinetics but failed to demonstrate durable benefit in PCa compared with other tumor types [218, 219, 220]. These experiences emphasize the need for biomarker‐guided trial design, adaptive dosing strategies, and stratification of PCa patients to ensure that therapeutic benefits are not masked by inter‐patient variability. Explicit acknowledgment of these limitations balances the optimism of preclinical evidence with the sobering realities of clinical translation.

While nanoparticles such as Abraxane or liposomal doxorubicin are widely studied in oncology, their clinical utility in PCa has been limited and often indirect. For instance, Abraxane demonstrated superior tumor uptake in breast and pancreatic cancers, yet efficacy in PCa remains modest; this section highlights the critical differences between general oncology applications and PCa‐focused advances, underscoring the need for disease‐tailored nanomedicine approaches [221, 222].

Table 1 summarizes representative nanoparticle systems evaluated in PCa and broader solid tumors, contrasting preclinical outcomes with clinical trial results. By including details such as nanoparticle type, therapeutic payload, target cancer, stage of clinical testing, efficacy observations, limitations, and references, this table provides a structured framework to assess both successes and failures. Such a comparative perspective underscores the urgent need for improved patient stratification, biomarker‐guided trial design, and mechanistic studies that better reflect the complexities of human tumors.

**TABLE 1:** Comparative analysis of nanoparticle systems in preclinical vs. clinical studies for PCa and solid tumors.

Nanoparticle system	Therapeutic payload	Target cancer	Preclinical efficacy	Clinical trial phase/outcome	Limitations observed	References
Liposomal doxorubicin (Doxil)	Doxorubicin	Ovarian, Breast, PCa	Strong tumor accumulation, reduced cardiotoxicity	FDA approved for ovarian/breast; limited benefit in PCa trials	Modest efficacy in solid tumors with poor vascularization	[14, 223]
Abraxane (albumin‐bound paclitaxel)	Paclitaxel	Breast, Pancreatic, PCa (tested)	Enhanced tumor uptake, superior to free paclitaxel in mice	Approved for breast, NSCLC, pancreatic; limited PCa efficacy	Toxicity and resistance in some patients	[224, 225]
BIND‐014 (PSMA‐targeted polymer NP)	Docetaxel	PCa, NSCLC	Potent tumor regression in xenografts	Phase II showed modest efficacy, not superior to docetaxel	Heterogeneous PSMA expression in patients	[14, 226]
CRLX101 (cyclodextrin‐based NP)	Camptothecin	Prostate, Renal, Ovarian	Sustained drug release, enhanced survival in mice	Mixed results in Phase II; limited PCa response	Drug resistance, variable pharmacokinetics	[227, 228]
Lipid nanoparticles (siRNA delivery)	siRNA against oncogenes	PCa, Liver cancer	Robust gene silencing in murine tumors	Early‐phase trials: delivery achieved, efficacy modest	Rapid clearance, immune activation	[229, 230]
Thermosensitive liposomes (lyso‐thermosensitive doxorubicin)	Doxorubicin	Liver, PCa (preclinical)	High release with mild hyperthermia, superior tumor control	Phase III in liver cancer ongoing; limited PCa testing	Tumor accessibility to hyperthermia limits use	[14, 231]
Gold nanoparticles (photothermal)	N/A (heat therapy)	PCa, Breast	Efficient tumor ablation in animal models	Early clinical safety established; limited efficacy data	Light penetration depth limits in deep tumors	[232, 233]
Polymeric Micelles (NK105)	Paclitaxel	Gastric, Breast	Improved solubility, reduced neurotoxicity	Phase III (breast) failed to show superiority	Drug release variability, modest efficacy	[234, 235]
Iron oxide nanoparticles	Imaging + Therapy	PCa, Brain tumors	Strong MRI contrast, drug delivery potential	Approved for imaging (Ferumoxytol); limited therapeutic success	Clearance, off‐target uptake in RES organs	[14, 236]
mRNA lipid nanoparticles	mRNA (therapeutic vaccines)	Solid tumors incl. PCa	Strong immune activation in mice	Early‐phase cancer vaccine trials ongoing	Immune‐related adverse events, limited efficacy so far	[237, 238]

## 6 Challenges and Future Directions

### 6.1 Overcoming Delivery Barriers in Hypoxic Tumors

The therapeutic and diagnostic components of nanomedicine struggle to reach their targets effectively in hypoxic tumors because hypoxic tumors have reduced blood vessel density and elevated interstitial pressure and modified metabolic behavior [117]. The solution of these obstacles represents a necessary condition to enhance both therapeutic outcomes and diagnostic imaging precision. Nanoparticles face two main obstacles which include restricted movement ability through tissue barriers combined with inadequate distribution of nanoparticles across the body [239]. The TME shows a disorganized network of blood vessels along with abundant ECM that creates barriers for nanoparticle movement and thus results in unequal medication distribution [240]. Nanoparticle accumulation in tumors through the EPR effect becomes compromised within hypoxic regions because they display reduced blood perfusion [240]. The circulating half‐life of nanoparticles decreases substantially due to systemic clearance mechanisms including both mononuclear phagocyte system (MPS) and renal filtration thus hindering their tumor site concentration [241]. Tumor uptake enhancement occurs through surface modification and stimuli‐responsive nanoparticles in combination with optimized nanoparticle size and shape as well as TME adjustment and specific transportation methods [242].

### 6.2 Safety, Cost, and Regulatory Considerations

Clinical translation of theranostic nanomedicine faces important safety concerns together with regulatory challenges as major obstacles. All nanoparticle‐based therapeutic products need to fulfill strict toxicity evaluation methods to evaluate their safety at short and long durations. The main safety issues regarding nanoparticle‐based therapeutics center around their compatibility with biological systems as well as their toxic effects and immune system responses and their ability to accumulate in the body and their additional impacts on healthy tissues [243]. Safety profiling requires understanding of the factors which determine how various nanoparticles will interact with non‐cancerous cells and organs. The specificity levels of nanoparticles should be high to avoid unwanted particle interactions.

A critical yet often underemphasized barrier to the clinical translation of TNPs is cost. The synthesis, large‐scale production, and quality control of multifunctional nanocarriers significantly increase manufacturing expenses compared with conventional drugs. Incorporating imaging agents, targeting ligands, and controlled release mechanisms further elevates costs, limiting accessibility and commercial feasibility. Additionally, regulatory compliance, extended toxicity testing, and good manufacturing practice (GMP) requirements contribute to prolonged development timelines and financial burdens. Therefore, achieving cost‐effective, scalable, and reproducible nanoparticle production remains essential to ensure equitable access and sustainable clinical adoption of nanomedicine in PCa management.

Despite encouraging preclinical safety data, clinical studies reveal unpredictable biodistribution, organ accumulation, and dose‐limiting toxicities. For example, metallic nanoparticles demonstrate efficient tumor ablation but can persist in organs with unclear long‐term clearance. Similarly, immune activation remains a barrier for repeated dosing of polymeric and lipid nanocarriers.

### 6.3 Food and Drug Administration (FDA) Approval Landscape for Theranostic Nanomedicine

TNPs undergo regulatory agency reviews from the FDA along with other agencies because these particles fulfill diagnostic and therapeutic functions. The regulatory agencies perform evaluations that include preclinical tests followed by clinical tests and operational quality checks and classification protocols. Safety, along with efficacy and biodistribution, is assessed through in vivo and in vitro lab tests, as well as Phase I–III clinical trials, which measure pharmacokinetics and assess toxicity and therapeutic benefit in human subjects.

Several complications in their regulatory process arise for TNPs since they function both as diagnostic tools and as treatments. The FDA of the United States, together with organizations like the European Medicines Agency (EMA), assesses these nanoparticles using rules for both pharmaceutical and device regulations [244]. Before actual human testing, thorough preclinical studies check for safety, spread of the drug in the body and results, then clinical trials are done in humans to look at pharmacokinetics, safety and the effects of the drugs [244]. Nanomedicines have been approved for use in cancer therapy, for example Doxil (liposomal doxorubicin), and in treating anemia and as an MRI contrast agent, an example being Ferumoxytol [245, 246]. There are regulatory problems associated with uneven size, ability to manufacture on a large scale and the durability over time of nanoparticles as used in biomedicine. In order to get past these obstacles, the FDA plans to set up standard test methods. New standards could help ensure both a quick licensing process and the effective use of dangerous nanotherapeutics.

Regulatory agencies remain cautious because TNPs blur the boundary between drugs and devices. The failure of PSMA‐targeted nanoparticles such as BIND‐014 to progress beyond phase II highlights that regulatory approval depends not only on efficacy but also on scalable manufacturing, consistent quality control, and predictable toxicity profiles.

### 6.4 Potential of Artificial Intelligence and Machine Learning in Theranostic Nanoparticle Development

Nanomedicine receives revolutionary enhancements from AI and ML that facilitate data‐based improvements of nanoparticle structures alongside prognostic capabilities for therapy results and speed up medication progress [55, 247]. AI computational models improve nanoparticle development rates through several functions, including nanoparticle property prediction and tumor biomarker along biological membrane interaction prediction and large‐scale property analysis for optimal design [247]. Computer programs using ML algorithms assist in searching through experimental data to find the most effective nanocarrier prospects [247]. Nanoparticle performance optimization results from multi‐objective optimization approaches, which manage various parameters. AI predictive solutions help medical practice through patient‐specific information processing while monitoring tumor responses and cutting down clinical testing expenses together with forecasting potential adverse medication effects.

Earlier studies have shown that through the use of DeepNano and AutoNano, ideal dimensions, surface charge and drug carrying ability of nanoparticles are predicted, making it simpler to send drugs with liposomes and dendrimers into cells [55, 106]. Nanocarrier candidates for targeted treatment of tumors are found using high‐throughput screening data and ML methods such as random forest and support vector machines [55]. AI‐generated nanoparticles are capable of crossing over the blood–brain barrier, an important barrier for brain tumor patients [248]. In situations where patients receive nanotherapeutics, clinical uses of IBM Watson and similar AI tools examine their personal genetics and scans to help figure out how tumors may react and to notice potential problems related to their treatment [249, 250]. Therefore, optimization of approaches like Pareto optimization have been applied to shape nanoparticles better so they enter cells more effectively and are less risky [251]. However, some challenges remain, for example, managing oxygen‐deprived parts of tumors with nanoparticles designed by AI and ensuring all AI‐based therapy products follow the rules set by regulatory bodies. Because of this, it is essential for nanotechnology engineers, computational biologists and oncologists to unite. Science and medical disciplines collaboration may help develop improved theranostic nanomedicine technology, giving patients safer, more effective cancer treatments.

AI‐informed and bioinspired PSMA nanomedicine examples include: (1) DNA‐scaffolded, PSMA‐liganded nanoparticles that precisely tune ACUPA ligand density to maximize uptake, an approach compatible with active‐learning optimization pipelines [252] (2) Cross‐linked PSMA‐targeted lipoic acid nanoparticles (cPLANPs) enabling pretargeted radiotherapy and synergistic treatment in metastatic CRPC [253] (3) PSMA‐targeted dendrimer (PD‐CTT1298) platforms for selective intracellular chemotherapy delivery [254] (4) Anti‐PSMA peptide–decorated exosome mimetics, generated by extrusion from engineered donor cells, achieving high‐yield, nanosized, PSMA‐avid vesicles [255] (5) Brusatol/docetaxel PSMA‐targeted polymeric NPs, with ligand density–dependent internalization, illustrating formulation knobs suited to ML optimization [208]. Together, these TNPs exemplify AI‐optimizable variables (ligand density, composition, release kinetics) and exosome‐mimicking strategies advancing PSMA‐directed PCa therapy.

While next‐generation nanomedicine strategies hold promise, their clinical translation for PCa remains limited by unresolved safety, biodistribution, cost, and regulatory challenges. A realistic path forward will require standardized toxicity testing, better models of long‐term biodistribution, immunogenicity, and harmonized regulatory frameworks must be addressed before widespread clinical adoption can occur.

## 7 Conclusion

In conclusion, TNPs represent a transformative strategy for PCa, combining targeted therapy with real‐time diagnostics. However, despite encouraging preclinical progress, clinical validation remains in its infancy. Limited efficacy of PSMA‐targeted formulations, modest trial outcomes with liposomal drugs, and persistent barriers such as hypoxia, tumor heterogeneity, and regulatory complexity highlight that widespread clinical adoption is not yet achievable. Moving forward, progress will depend on (i) biomarker‐driven patient stratification, (ii) integration of AI for nanoparticle design, (iii) standardized regulatory pathways, and (iv) interdisciplinary collaboration to translate laboratory innovation into patient benefit. A balanced perspective therefore acknowledges both the rich promise and the unresolved limitations of theranostic nanomedicine in PCa. Hence, future work should especially prioritize synergizing nanoparticle‐based PSMA targeting with ADT, PARP inhibition, and immunotherapy to address the unique therapeutic challenges of PCa.

## Author Contributions

Conceptualization: D.E.U., I.J.A., E.U.A., G.E.E.; writing original draft preparation: D.E.U., W.A.O., I.B., O.P.‐C.U., A.O.I.; writing review and editing, all authors; and all authors have read and agreed to the final version of the manuscript.

## Funding

The authors have nothing to report.

## Disclosure

*Institutional Review Board Statement*: The authors have nothing to declare.

## Ethics Statement

The authors have nothing to report.

## Consent

The authors have nothing to report.

## Conflicts of Interest

The authors declare no conflicts of interest.

## Supporting information


**Data S1:** Supporting Information.

## Data Availability

Data sharing not applicable to this article as no datasets were generated or analysed during the current study.
