# Supplementary material for: Theranostic Nanoparticles in Prostate Cancer: Disrupting Hypoxia‐Induced Glycolysis by Targeting Hypoxia‐Inducible Factor‐1 Alpha and Downstream Metabolites
Source: Cancer Med. 2026 Jan 11;15(1):e71519. doi: 10.1002/cam4.71519 (PMC12790959; doi:10.1002/cam4.71519)
Supplement: Supplementary file 1 — Data S1: Supporting Information. [file CAM4-15-e71519-s001.docx]

**Table 1S: Chemically Stimulated Hypoxia-Responsive Nanomaterials**

| **Nanomaterial System** | **Hypoxia-Sensitive Moiety** | **Activation Mechanism** | **Application** | **Nanocarrier Type** | **Advantages** | **Reference** |
| --- | --- | --- | --- | --- | --- | --- |
| Liposomes with nitroimidazole | Nitroimidazole | Enzymatic reduction → drug release | Tumor-targeted chemotherapy | Liposome | High specificity, bioreduction-sensitive | [128,129] |
| Polymeric micelles with azo linkers | Azo group (–N=N–) | Azoreductase-mediated cleavage | Colon-targeted therapy | Micelle | Enzyme-triggered, stable under normoxia | [117, 130] |
| Gold nanoparticles with quinone linkers | Quinones (e.g., naphthoquinone) | Redox reduction to hydroquinones | Imaging and drug delivery | Gold NP | Redox-responsive, stable and biocompatible | [131, 132] |
| Dendrimers with sulfonamide | S[55]ulfonamide linker | pH/hypoxia dual cleavage | Hypoxia-targeted drug delivery | Dendrimer | Dual-stimuli responsive, enhanced selectivity | [55, 133] |
| Mesoporous silica nanoparticles | Diazobenzene linker | Hypoxia-triggered degradation | Prodrug delivery in hypoxic tumors | MSNs | Customizable pores, tunable release | [134, 135] |
| Polymeric prodrugs | Nitroaromatic group | Bioreductive cleavage to release active drugs | Solid tumor therapy | Polymeric carrier | Low immunogenicity, long circulation | [136, 137]*.* |
| Smart hydrogels | Quinone-methide linkage | Hypoxia-triggered depolymerization | Localized drug delivery | Injectable hydrogel | High payload, minimal systemic exposure | [138, 139] |

**Table 2S: Clinical and Preclinical Advances in Theranostic Nanoparticles for Prostate Cancer: Key Findings, Challenges, and Future Prospects**

| **S/N** | **Category** | **Subcategory** | **Key Findings** | **Implications** | **Challenges** | **Future Prospects** | **Type of Nanoparticles** | **References** |
| --- | --- | --- | --- | --- | --- | --- | --- | --- |
| **1** | In Vivo Studies | Theranostic Nanoparticles | Enhanced tumour targeting and reduced toxicity | Improved therapeutic index | Biocompatibility and long-term safety concerns | Personalized delivery strategies | Liposomes, Polymeric NPs | [29, 155] |
| **2** | In Vitro Studies | Theranostic Nanoparticles | Increased cellular uptake and drug retention | Higher treatment efficacy | Need for better in vitro models | Optimization of nanoparticle design | Polymeric NPs, Dendrimers | [224, 225] |
| **3** | Efficacy in Prostate Cancer | Animal Models | Significant tumour regression observed | Potential for clinical translation | Variability in tumour respons | Large-scale preclinical validation | Liposomes, Metallic NPs | [226, 227] |
| **4** | Biocompatibility | Safety Profile | Some nanoparticles show minimal cytotoxicity | Safer therapeutic options | Need for extensive toxicity profiling | Development of biodegradable nanocarriers | Polymeric NPs, Lipid NPs | [228, 229] |
| **5** | Toxicity Concerns | Long-term Effects | Potential accumulation in organs | Risk assessment for clinical applications | Clearance mechanisms not well understood | Engineering biodegradable nanoparticles | Metallic NPs, Carbon-based NPs | [55, 163] |
| **6** | Drug Resistance | Tumour Adaptation | Resistance observed in prolonged treatments | Need for combination therapies | Complex molecular mechanisms involved | Use of multi-functional nanocarriers | Polymeric NPs, Hybrid NPs | [230, 231] |
| **7** | Tumour Heterogeneity | Variability in Response | Different patient responses to same treatment | Need for patient-specific therapies | Limited biomarkers for stratification | Integration with precision medicine | Liposomes, Exosomes | [163, 163] |
| **8** | Clinical Trials | Current Landscape | Several nanoparticle formulations in trials | Growing interest in nano-based therapies | Limited long-term clinical data | Accelerated regulatory pathways | Polymeric NPs, Liposomes | [55, 163] |
| **9** | Personalized Nanomedicine | Tailored Approaches | Emerging strategies for patient-specific use | Increased effectiveness | High cost and complexity | AI-driven and biomarker-based therapy selection | Exosomes, Polymeric NPs | [232, 233] |
| **10** | Future Directions | Innovations in Theranostics | Advanced imaging and drug delivery systems | Enhanced real-time monitoring | Need for interdisciplinary collaboration | Smart nanoparticles with adaptive properties | mart NPs, Quantum Dots | [55, 234] |

**NPs= Nanoparticles**
